# Acute L-Carnitine Supplementation Does Not Improve CrossFit^®^ Performance: A Randomized, Double-Blind, Placebo-Controlled Crossover Study

**DOI:** 10.3390/nu17172784

**Published:** 2025-08-27

**Authors:** Asli Devrim-Lanpir, Lucero Salazar Velasco, Fanny Guadalupe Ramirez Lara, Azucena Ojeda Sanchez, Rachel Kimble, Reza Zare, Fatma Esra Gunes, Beat Knechtle, Katja Weiss, Thomas Rosemann, Katie Heinrich

**Affiliations:** 1 School of Health and Human Performance, Dublin City University, D09 V209 Dublin, Ireland; 2 Department of Nutrition and Dietetics, Faculty of Health Sciences, Istanbul Medeniyet University, 34862 Istanbul, Turkey; asli.devrim@medeniyet.edu.tr (A.D.-L.); fatmaesra.gunes@medeniyet.edu.tr (F.E.G.); 3 Faculty of Higher Studies Zaragoza, National Autonomous University of Mexico, Mexico 09230, Mexico; lucerosv@comunidad.unam.mx (L.S.V.); lupera1000@gmail.com (F.G.R.L.); azucenaojedasan@yahoo.com.mx (A.O.S.); 4 Division of Sport and Exercise Science, School of Health and Life Sciences, University of the West of Scotland, Blantyre G72 0LH, UK; rachel.kimble@uws.ac.uk; 5 SRH Campus Hamburg, Berlin University of Applied Sciences, 20095 Hamburg, Germany; reza.zare.official73@gmail.com; 6 Medbase St. Gallen Am Vadianplatz, Vadianstrasse 26, 9001 St. Gallen, Switzerland; 7 Institute of Primary Care, University of Zurich, Pestalozzistrasse 24, 8091 Zurich, Switzerland; katja@weiss.co.com (K.W.); thomas.rosemann@usz.ch (T.R.); 8 Department of Kinesiology, Kansas State University, Manhattan, KS 66506, USA; kmhphd@ksu.edu; 9 Department of Research and Evaluation, The Phoenix, Manhattan, KS 66502, USA

**Keywords:** L-carnitine, CrossFit^®^, exercise performance, high-intensity training, supplementation

## Abstract

Background: L-carnitine supplementation is thought to enhance exercise performance, particularly in moderate and high-intensity activities, but evidence supporting this is mixed. This study aimed to assess whether acute L-carnitine tartrate supplementation could improve CrossFit^®^ performance, specifically during the “Cindy” workout, a high-intensity exercise protocol. Methods: In a randomized, double-blind, placebo-controlled crossover design, 20 trained male recreational CrossFit^®^ athletes completed the “Cindy” workout within a 20 min period after ingesting either 3 g of L-carnitine tartrate or a placebo 90 min before exercise. Performance was measured by total repetitions completed. Secondary outcomes included ratings of perceived exertion (RPE), gastrointestinal issues, and blood pressure (BP) measurements. Results: The results showed that L-carnitine supplementation did not significantly affect the number of repetitions performed (202.4 ± 69.9 vs. 204.5 ± 78.8, *p* = 0.810) compared to the placebo. There were also no significant differences in RPE (6.3 ± 1.5 vs. 6.9 ± 1.4, *p* = 0.180) or BP changes between groups. However, 10% of participants reported difficulty sleeping after L-carnitine supplementation. Conclusions: The findings suggest that 3 g of L-carnitine tartrate does not enhance CrossFit^®^ performance in recreational athletes. Further research is needed to clarify its potential benefits, especially with larger samples and consideration of factors like sex and carbohydrate co-ingestion.

## 1. Introduction

Carnitine has gained significant attention as a weight loss supplement due to its proposed role in facilitating fat oxidation. Widely marketed as a “fat burner,” L-carnitine is believed to enhance the aerobic contribution to exercise by increasing fat oxidation, thereby promoting the conversion of fat into energy [1]. This has led to its popularity not only as an ergogenic aid but also as a supplement aimed at reducing fat mass and increasing muscle mass [2]. Although some studies suggest that carnitine can improve exercise performance, recovery, and reduce muscle soreness [3,4], others report no significant benefits [5,6]. Most of the research has focused on endurance athletes, [5,7] with limited studies examining the effects on other sports disciplines, including CrossFit^®^.

Carnitine, a naturally occurring amino acid derivative, is pivotal in energy production by transporting long-chain fatty acids into the mitochondrial matrix for beta-oxidation [8]. This process is essential for producing ATP, the primary energy currency of the cell, particularly during prolonged and high-intensity exercise [9]. Additionally, carnitine may reduce blood lactate accumulation by interacting with acetyl-CoA to form acetyl-carnitine and CoA, thereby decreasing lactate levels [10]. Furthermore, in vitro and in vivo studies have suggested carnitine’s role as an antioxidant, which could mitigate exercise-induced muscle damage and enhance recovery [11]. Given these factors, L-carnitine supplementation may offer potential benefits by influencing various physiological and metabolic pathways, potentially enhancing exercise performance in both moderate and high-intensity activities, provided that increases in muscle carnitine content can be achieved [4]. Carnitine has two stereoisomers: L-carnitine, the biologically active form essential for transporting long-chain fatty acids into mitochondria for energy production, particularly during intense exercise, and D-carnitine, which is inactive and can inhibit L-carnitine’s function [12]. Due to its effectiveness and potential roles on exercise performance, metabolism, and energy production, L-carnitine is the form used in therapeutic or research settings [13]. Despite its popularity, scientific evidence on the efficacy of carnitine supplementation remains mixed [5,6,14]. Acute L-carnitine/L-carnitine-tartrate protocols most often use 3–4 g ingested 60–90 min pre-exercise and have shown benefits primarily in high-intensity tasks [4]. Regarding high-intensity exercise performance (≥80% VO_2_ max), the potential impact of carnitine in maintaining the acetyl-CoA/CoA ratio and thereby reducing lactate accumulation and decreasing inflammation is particularly important during maximal and supramaximal exercises [4]. Since CrossFit^®^ training includes these types of exercise regimens [15], L-carnitine supplementation could potentially improve performance. However, to the authors’ knowledge, no study has examined this interaction.

The recent surge in high-intensity functional training, notably driven by CrossFit^®^, aims to enhance multiple domains of physical fitness by exposing athletes to various modes of exercise, such as endurance and resistance training, within and between sessions for durations ranging from 2 to 60 min at relatively high intensities [16]. This training method has been experiencing significant growth, with over 14,000 affiliated gyms established across 150 countries [17]. CrossFit^®^, exemplified by its annual competition, the CrossFit^®^ Games, epitomizes this training methodology. During CrossFit^®^ sessions, athletes may perform up to 700 repetitions in a 20 min workout, demonstrating the sport’s demanding nature. CrossFit^®^ athletes typically exhibit high levels of health- and skill-related physical fitness aspects, with significant correlations found between CrossFit^®^ performance and aerobic capacity, muscular strength (both upper- and lower-body), and power [18]. CrossFit^®^ training includes a mix of physical exercises such as weightlifting, powerlifting, sprints, plyometrics, calisthenics, gymnastics, and running [16,18]. Despite the rigorous and varied nature of CrossFit^®^ training, there is limited evidence from the sports nutrition community regarding the utility of dietary supplementation to enhance CrossFit^®^ performance [19]. While studies on supplement use and CrossFit^®^ performance have examined the effects of nitrate [20], caffeine [21,22], betaine [23,24], sodium bicarbonate [25], and beta-alanine [19,26], there are currently no studies investigating the effects of carnitine, either in acute or chronic administration.

Given the current gaps in the literature, this study aims to investigate the acute effects of carnitine supplementation on CrossFit^®^ performance. The primary objective is to determine whether carnitine supplementation can enhance exercise performance, measured by the total number of repetitions in recreational CrossFit^®^ athletes. A secondary aim was to examine ratings of perceived exertion (RPE) and blood pressure (BP) measurements. In addition, percent changes in performance between sessions are calculated to identify whether the learning effect has any effects on the ergogenic effect of L-carnitine.

## 2. Materials and Methods

### 2.1. Participants

Twenty trained young male adults, aged 18–35 years (25.2 ± 4.1 years), participated in the study (mean ± SD: with an average height of 170 ± 6 cm, body mass of 69.8 ± 12.2 kg, and BMI of 23.8 ± 3.7 kg/m^2^). Participants were required to have at least six months of CrossFit^®^ experience and have completed the “Cindy” routine. Exclusion criteria included any diagnosed illnesses, smoking, recent use of medications or supplements (within the last three months), family history of seizures, or musculoskeletal injuries. The study was conducted in a single CrossFit^®^ club at the Faculty of Higher Studies of Zaragoza to minimize the effects of varying training programs across different clubs. Participants were selected through convenience non-probabilistic sampling, applying strict inclusion and exclusion criteria to ensure a representative sample of CrossFit^®^ athletes who had previously completed the Cindy workout. All participants were informed about the study procedures and provided written informed consent prior to participation. The study received approval from the Research Ethics Committee of National Autonomous University of Mexico (Approval Reference: FESZ/CEI/31/23) and was conducted in accordance with the Declaration of Helsinki. Participants were enrolled between 27 November and 27 December 2023, and because every outcome and adverse-event check was completed during or within ~30 min of each workout, follow-up concluded on the day of each participant’s second visit; the last study procedure was finished on 27 December 2023. This study was registered in the ISRCTN registry on 29 January 2025 under registration number ISRCTN14089170.

### 2.2. Experimental Design

**Figure 1:**
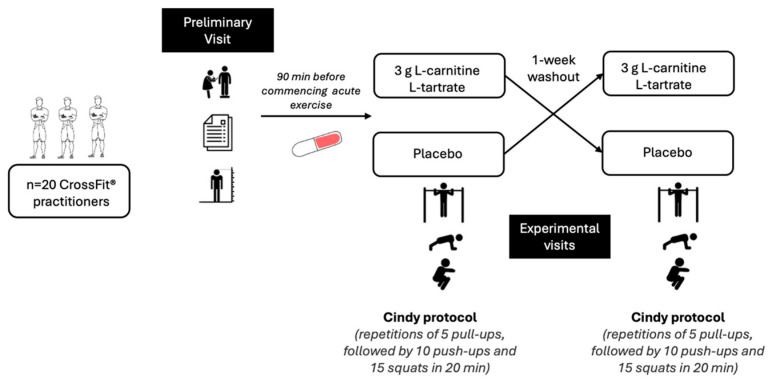
Schematic of the Study Design.

The study employed a randomized, double-blind, placebo-controlled, crossover design (Figure 1). To preserve masking, an independent pharmacist prepared and dispensed all supplements so that both participants and investigators were unaware of assignment. Outcome assessors were kept separate from randomization and remained blinded to allocation throughout data collection and analysis. An independent pharmacist prepared indistinguishable L-carnitine and placebo capsules and kept the randomization key, so the bottles, labels and contents looked the same to everyone. Consequently, participants, exercise supervisors, outcome assessors and data analysts all worked with blind group codes (A/B) that were revealed only after primary analyses were complete. An independent pharmacist handed each participant the coded capsule (3 g L-carnitine L-tartrate or microcrystalline-cellulose placebo), supervised ingestion with water 90 min before every workout, and dose logs showed every capsule was taken as scheduled, confirming 100% fidelity and adherence in both periods. We did not collect end-of-study allocation guesses; therefore, the success of blinding could not be formally quantified. While participants, exercise supervisors, outcome assessors and data analysts were blinded to the supplement allocation, participants were not blinded to the in-session repetition count inherent to as many rounds and reps as possible (AMRAP) scoring; judges tallied repetitions to enforce movement standards and ensure objective scoring. Participants were instructed to abstain from alcohol, caffeine, and nicotine for 24 h prior to testing. In addition, they were instructed to maintain their usual training throughout the study but to abstain from exhausting exercise for the 24 h preceding each visit. Visits were separated by a 7-day washout, which exceeds ~9–10 elimination half-lives of L-carnitine (t^½^ 17.4 h) and is consistent with prior crossover studies [27,28], making pharmacological carryover after a single acute dose unlikely. They were advised to maintain their usual diet, stay adequately hydrated [29], avoid consuming food three hours before testing, and continue with their regular training regimen throughout the study. Anthropometric measurements (height (using a Stadiometer (SECA, Hamburg, Germany)), body mass (using a calibrated scale (Tanita 780, Tokyo, Japan) and body circumference measurements using the ISAK measurement procedures) [30] were measured during the initial visit.

Before the second visit, participants were randomly divided into two groups using an online random generator [31]. Participants took an indistinguishable capsule (double-blinded) containing either 3 g of L-carnitine (L-carnitine L-tartrate) or placebo (microcrystalline cellulose) ninety minutes before commencing the study exercise protocol. The timing for supplementation was chosen based on recommendations from previous studies on carnitine and exercise performance [4]. Both supplements were kept in visually identical capsules and containers.

The workout “Cindy” was selected as it is a standardized CrossFit^®^ routine, well-documented in the literature [32]. Briefly, participants completed as many rounds as possible within a 20 min period, following CrossFit^®^ movement standards, consisting of five pull-ups, 10 push-ups, and 15 air squats in each round. The specific movement standards for the exercises are as follows:

Pull-ups: Participants were permitted to use kipping. Each athlete selected a pull-up style and grip at the first visit and replicated the same style, grip, and bar height at the second visit. Athletes maintained a hang from the bar without the feet touching the ground during repetitions; assistance (e.g., stepping on a box) was used only to mount the bar and did not contribute to the count. Certified judges monitored execution, gave immediate ‘no-rep’ feedback for any repetition not meeting standards, and tallied only valid repetitions.

Push-ups: Participants are required to perform push-ups on their toes, ensuring a straight body alignment throughout the movement. They must lower their bodies until their chest contacts the ground and then extend their arms fully to complete each repetition.

Air Squats: Participants must achieve full extension of the knees and hips at the top of each repetition, and ensure that the hip crease descends below the knee at the bottom of each repetition.

Performance was evaluated based on the total number of repetitions completed within the 20 min period. Evaluators with CrossFit^®^ Level 1 or 2 certificates were responsible for verbally counting repetitions. Prior to data collection, all evaluators held a standardization meeting to review the written movement standards and ‘no-rep’ criteria and practiced with example repetitions to align judgments. During testing, judges counted only valid repetitions and provided immediate ‘no-rep’ feedback for any deviation. When scheduling allowed, the same judge assessed both visits for a given participant to enhance within-subject consistency. Each trial was performed individually (one athlete per station) with a dedicated certified judge; no other athletes were present, and no visible clock or music was used to avoid external pacing or competition cues. Sessions were scheduled based on participant availability and time of day was not standardized. The study exercise protocol was conducted indoors with room temperature set at 22 °C.

Blood pressure measurements, including diastolic (DBP) and systolic (SBP), were taken on the left arm with participants seated in accordance with the American College of Sports Medicine recommendations [33]. Measurements were recorded prior to each session after a 10 min rest period and again 15 min following the exercise session.

Participants completed the same study procedure when they came to the laboratory after a 7-day wash-out period. Everything was set identically except the supplement administered. A post-exercise online survey was completed after each exercise session. The survey included questions on whether they perceived any effect after supplement administration (yes/no), the rating of perceived exertion (RPE) achieved during the workout on a scale of 1–10, and questions whether they experienced any side effects (nausea or vomiting, abdominal pain, diarrhea, fever, excessive sweating, alterations in the perception of flavors in foods or drinks, heartburn, fishy smelling sweat, muscular weakness, metallic flavor in the mouth, difficulty sleeping) after exercise (yes/no). Participants completed the post-exercise online survey ~30 min after finishing the session, at which time they reported session RPE (Borg scale) and answered questions on adverse symptoms [34].

The prespecified primary outcome was the total number of repetitions completed during the 20 min “Cindy” workout. This variable was analyzed as the final count recorded at workout completion and summarized as the mean ± standard deviation (SD) across participants.

Secondary outcomes were: (i) rating of perceived exertion (Borg CR-10 scale) obtained immediately after exercise, analyzed as final scores and expressed as mean ± SD; (ii) systolic and diastolic blood pressure measured after a 10 min seated rest (baseline) and again 15 min post-exercise, analyzed as the change from baseline and reported as mean ± SD; and (iii) gastrointestinal or other adverse symptoms captured in an online questionnaire completed within ~30 min of the session, reported as the number and percentage of participants experiencing each symptom.

### 2.3. Data Analysis

An a priori power analysis was conducted for a repeated-measures Analysis of Variance (ANOVA) to evaluate within-between interactions based on a study evaluating the impact of acute caffeine supplementation on CrossFit^®^ performance [21]. The analysis, which assumed an expected effect size (ES) of 0.28, an alpha level of 0.05, a correlation of 0.65 between repeated measures, and a statistical power of 80%, determined that a sample size of 20 participants was required. This power analysis was performed using G*Power (version 3.1; Düsseldorf, Germany).

Statistical analyses were conducted in SPSS v29.0 (IBM, Armonk, NY, USA). Results are reported as mean ± SD. Because all 20 participants completed the outcomes, we used a complete-case approach with no imputation. Distributional assumptions were checked with Kolmogorov–Smirnov and Shapiro–Wilk tests. Paired-samples *t*-tests compared the carnitine and placebo conditions and evaluated a possible between-session (learning) effect. Percentage change between sessions was computed as ((Session 2 − Session 1)/Session 1) × 100, and percentage change between conditions as ((Carnitine − Placebo)/Placebo) × 100. A two-way repeated-measures ANOVA (supplement × time) was used to analyze the CrossFit^®^ exercise outcomes following carnitine and placebo. Statistical significance was set at *p* < 0.05. Effect magnitude for the carnitine–placebo contrast was expressed as Cohen’s d, interpreted as large (>0.8), moderate (0.5–0.8), small (0.2–0.5), and trivial (<0.2) [35].

## 3. Results

**Figure 2:**
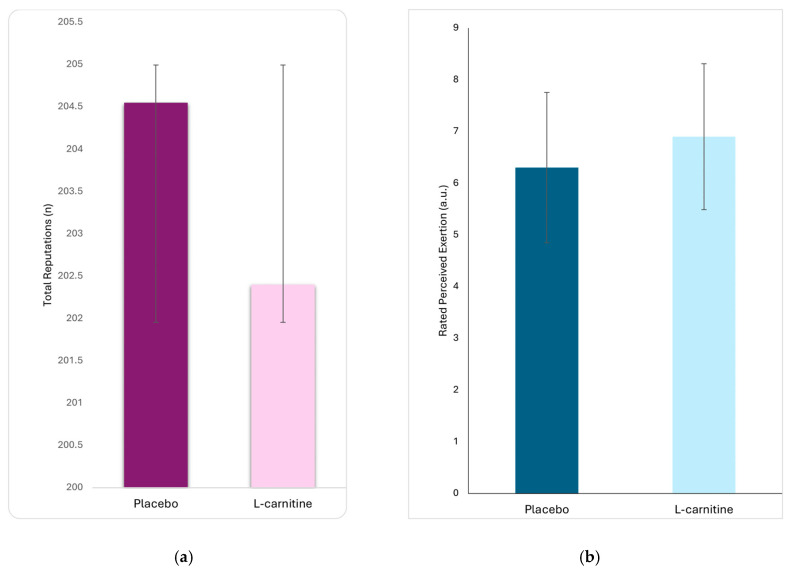
Comparison of performance parameters between the carnitine and placebo groups: (**a**) total repetitions and (**b**) rate of perceived exertion (RPE).

Twenty athletes were randomized (11 to begin with L-carnitine and 9 with placebo), every participant swallowed the allocated capsule at each visit, completed the 20-min Cindy workout twice, and all 20 were therefore included in the primary-outcome analysis. All 11 athletes allocated to L-carnitine and all 9 allocated to placebo completed both study visits; no participants were lost to follow-up, withdrew, or were excluded for any reason, so the analyzed numbers matched the randomized numbers. Among the performance measurements, no significant differences were observed between the carnitine and placebo groups in the total number of repetitions performed (202.4 ± 69.9 vs. 204.5 ± 78.8, t = 0.243, *p* = 0.810) (Figure 2a) and ratings of perceived exertion (6.3 ± 1.5 vs. 6.9 ± 1.4, t = 1.39, *p* = 0.180) (Figure 2b). Between conditions, the mean paired difference was +2.85 reps (Carnitine-Placebo; 95% CI −15.6 to +21.3), t (19) = 0.323, *p* = 0.750; the paired standardized effect was Cohen’s d₍z₎ = 0.07 (trivial).

Table 1 shows the total number of repetitions during the first and second sessions, percent changes in performance between sessions applied (learning effect) and percent changes of repetitions based on the supplement applied. No meaningful learning effect was detected between the first and second sessions (211.0 ± 74.4 vs. 195.9 ± 73.9, t = 2.09, *p* = 0.08, Cohen’s *d* = 0.20 (small)). Additionally, no significant treatment effect was observed between the carnitine and placebo groups (F = 0.606, *p* = 0.674) (Table 1). Post hoc, we classified individual changes (Carnitine- Placebo) using a ±10% threshold and, to temper percentage inflation at low baselines, an absolute cutoff of ≥20 repetitions (~10% of the cohort mean ~200 reps; ~2/3 of a Cindy round; ~0.5 SD of the within-person differences). By these criteria, 6/20 (30%) improved ≥10%, 4/20 (20%) declined ≤−10%, and 10/20 (50%) were within ±10%; 5 athletes improved by ≥20 reps and 5 declined by ≥20 reps. The median %Δ was −0.63% and the 10% trimmed mean %Δ was +0.46%, whereas the mean %Δ was +13.53% due to one extreme positive responder.

Individual performance during the Cindy workout by condition. Columns show per-participant totals and within-subject change (Δ = Carnitine − Placebo; positive values favor Carnitine). Rows are sorted by Δ (largest to smallest). %Δ = 100 × Δ/Placebo.

Out of 20 subjects, ten (50%) perceived the effect of the supplement during the carnitine trial, while six (30%) perceived an effect during the placebo trial. The remaining four participants (20%) reported no perceived effect in either condition. Their session-RPE values were similar to the cohort distribution in both trials. Two out of 20 subjects (10%) supplemented with carnitine reported difficulty sleeping, while abdominal pain (*n* = 1) and excessive sweating (*n* = 1) were reported following placebo administration.

**Figure 3:**
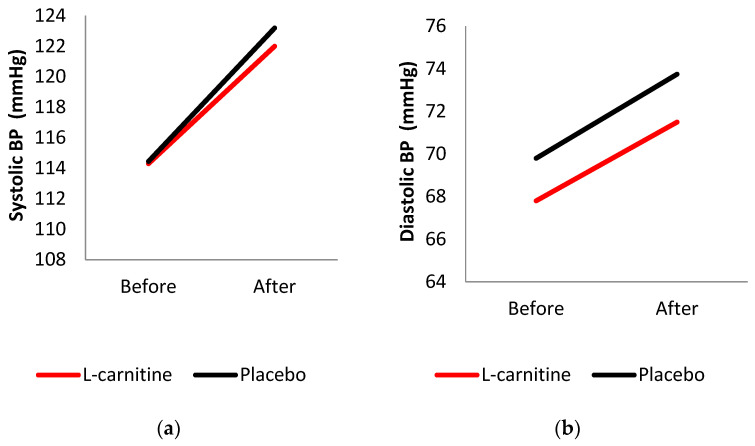
Cardiovascular parameters. (**a**) Systolic blood pressure (mmHg) before and after the intervention in the L-carnitine and placebo groups; (**b**) Diastolic blood pressure (mmHg) before and after the intervention in the L-carnitine and placebo groups. (Red line = L-carnitine; black line = placebo).

Figure 3 represents some cardiovascular parameters, including systolic and diastolic BP. No meaningful differences were detected between groups before or after supplementation.

## 4. Discussion

This research examined the potential efficacy of L-carnitine supplementation on exercise performance, as determined by Cindy protocol, in recreational CrossFit^®^ athletes, comparing it to a placebo. Our hypothesis posited that L-carnitine administration before exercise would enhance exercise performance compared to the placebo. However, the outcomes contradicted this hypothesis, indicating no significant efficacy of L-carnitine on performance compared to the placebo group.

To our knowledge, this is the first study to evaluate the effects of acute L-carnitine supplementation on CrossFit^®^ performance, as measured by the “Cindy” workout. The CrossFit^®^ “Cindy” protocol is a unique multi-joint workout that presents a high-volume muscular endurance challenge over an extended period (20 min) with minimal rest [36]. As this workout demands muscular endurance, power, and resistance capacity [32,36], in this study, it was hypothesized that the potential effectiveness of L-carnitine on CrossFit^®^ performance could be related to its ability to regulate the acetyl-CoA/CoASH ratio in the mitochondria, thereby increasing the rate of carbohydrate delivery during endurance performance, and/or improving recovery between repetitions by increasing antioxidant capacity or reducing lactate accumulation during high-load exercise. However, contrary to our hypothesis, findings indicated that L-carnitine supplementation (3 g L-carnitine) did not significantly enhance “Cindy” workout performance in recreational CrossFit^®^ athletes.

These results align with the current literature on acute L-carnitine administration, which highlights the inconsistency of findings regarding its efficacy on performance in athletic or physically active individuals [4]. While studies on acute L-carnitine supplementation have shown that short-term supplementation at doses of 2–4.5 g significantly enhances peak and mean power output during repeated high-intensity cycle sprints, reduces blood lactate accumulation in resistance-trained male subjects [37], and reduces blood lactate levels, increases blood glucose levels, and enhances both aerobic (VO_2_ max) and anaerobic (mean and maximum power) performance in elite male artistic gymnasts [38], another study indicated that a 2 g dose of L-carnitine taken 2 h before exhaustive exercise did not improve performance, recovery, or physiological responses in a second constant-load exercise test performed 3 h after the first test, compared to a placebo [5]. One possible explanation for these results is the challenge of achieving the desired ergogenic effects of L-carnitine supplementation. Increasing muscle carnitine content has proven to be quite challenging [14]. Studies suggesting a positive impact of L-carnitine supplementation emphasize the importance of creating hyperinsulinemia to increase muscle L-carnitine content [39]. Therefore, it is recommended to co-ingest carbohydrates (approximately 80 g) with L-carnitine supplements, as hyperinsulinemia induced by co-ingesting whey protein has been ineffective in boosting muscle L-carnitine content [39]. Since no carbohydrate supplementation was applied in the present study, future research on CrossFit^®^ performance and L-carnitine might consider this approach. However, it is important to note that CrossFit^®^ athletes often follow restrictive diets like Paleo and Zone diets and use ergogenic supplements to lose body fat or weight for performance and esthetic reasons [26]. As demonstrated in studies where carbohydrates were co-ingested with L-carnitine [39], this approach may lead to weight gain. Therefore, the potential for weight gain should be carefully considered before implementing this strategy.

A systematic review and meta-analysis on L-carnitine and blood pressure indicated that L-carnitine attenuated diastolic blood pressure without affecting systolic blood pressure. This effect can be explained by the role of cardiomyocytes in regulating energy metabolism and the functions of cardiac fibroblasts, as well as its impact on nitric oxide metabolism [40]. However, in contrast to these findings, no significant changes in diastolic or systolic blood pressure were observed during exercise between the L-carnitine group and the placebo group. Further studies are required to elucidate these findings.

Although studies on L-carnitine supplementation have reported some gastrointestinal side effects, including diarrhea, nausea, vomiting, and stomach cramps [41], the present study showed no gastrointestinal problems with L-carnitine administration. However, 10% of participants experienced difficulty sleeping after L-carnitine supplementation. This finding is surprising, as some studies have indicated that L-carnitine can decrease fatigue and improve mood and sleep quality [42]. Further research is needed to clarify this interaction.

Our research offers several notable advantages. Its design as a double-blinded, placebo-controlled, cross-over study allowed participants to serve as their own controls, thereby accurately determining the impact of the acute supplementation. The present research evaluated sports performance using the “Cindy” protocol, which is widely recognized in the literature for assessing CrossFit^®^ performance. No learning effect was detected between the two applications of the Cindy protocol during the study, indicating that the learning effect did not overshadow the ergogenic effect of L-carnitine.

However, there are a few limitations in our study that should be acknowledged. No familiarization sessions were conducted before the experimental protocol. Nonetheless, since only CrossFit^®^ athletes with experience in the Cindy protocol were included and no learning effect was detected, the absence of familiarization sessions likely did not significantly affect the results. We did not quantify plasma or intramuscular carnitine after dosing. Insulin stimulation (via carbohydrate intake or insulin infusion) facilitates carnitine transport into skeletal muscle following oral supplementation; chronic co-ingestion with carbohydrate over weeks has been shown to increase muscle carnitine content and influence fuel selection, whereas acute pre-exercise protocols, even when combined with carbohydrate, generally do not meaningfully modify substrate use, consistent with an insufficient rise in the muscle carnitine pool. Our acute 3 g L-carnitine tartrate protocol without controlled carbohydrate co-ingestion therefore may have provided inadequate intramuscular availability, offering a plausible explanation for the absence of ergogenic effects. Another limitation is that individual responses varied widely. Because these analyses were post hoc and not pre-registered, they are hypothesis-generating and should be interpreted cautiously. Potential contributors include uncontrolled pre-exercise nutrition, time of day, week-to-week training, and normal performance variability; future pre-registered studies with larger samples should model individual treatment effects and test predictors of responsiveness. The experimental protocols were conducted with participants alone, without a visible clock or music, to maintain internal validity. However, CrossFit^®^ gyms typically have music, many people, and a visible clock, which could influence real-life performance [43,44]. First, participants were necessarily aware of their in-session repetition count during the Cindy AMRAP, which could influence pacing or motivation. Although our randomized, double-blind, crossover design and the absence of external time cues (no visible clock, no music, individual sessions) mitigate systematic bias, we acknowledge this as a limitation. In addition, sleep duration and quality were not measured, which could be important determinants of exercise performance in CrossFit^®^ athletes [45]. Although a 24 h exhausting exercise restriction was prescribed to eliminate the potential impact of recent training sessions, training logs during the experimental period were not collected. This could affect the results, as the weekly training volume might influence performance due to potential overtraining or fatigue/delayed onset muscle soreness from other training sessions. Randomized order should balance any period-related changes across supplement conditions, and the observed session effect was small and in the direction of lower performance at Session 2, arguing against systematic training-induced improvements between visits. We did not administer a formal blinding-check (participant allocation guesses), which prevents quantifying blinding efficacy. We did not collect nutrition or training diaries across the study period. Consequently, between-visit differences in pre-exercise carbohydrate and caffeine intake, hydration, and week-to-week training load may have affected performance and increased within-subject variability. Although randomization, blinding, a 24 h abstention from exhausting exercise, and a 7-day wash-out should balance such variability across conditions (likely attenuating rather than inflating any effect), this remains a source of potential confounding. However, because the study used a randomized, double-blind, placebo-controlled crossover design, any uncontrolled dietary effects should be balanced across conditions and most likely attenuate, rather than inflate, between-condition differences. We did not standardize the time of day for testing, which may introduce circadian variability in performance; however, randomized order and the crossover design should balance such effects across conditions. Although our a priori power targeted a small within-subject effect (d₍z₎ = 0.28) based on the prior Cindy crossover study [21], the modest *n* = 20 and high variability in Cindy performance limit precision. The observed effect on repetitions was trivial (d₍z₎ ~ 0.07) with a wide 95% CI, so very small benefits or harms cannot be excluded, although a practically meaningful improvement under this acute protocol appears unlikely. Lastly, the study included trained men only; therefore, results should not be generalized to women or to the broader CrossFit^®^ community. Potential sex-specific differences in performance and physiological responses mean that ergogenic effects (or lack thereof) could differ in women. Lastly, we administered an acute dose of 3 g L-carnitine, as suggested by literature, to improve athletic performance. However, it remains unclear whether variations in body composition may influence an individual’s response to L-carnitine supplementation.

## 5. Conclusions

A 3 g dose of L-carnitine tartrate taken 90 min prior to the “Cindy” workout did not impact the number of repetitions performed by recreational CrossFit^®^ athletes. Moreover, there were no significant differences in perceived exertion (as measured by RPE) or blood pressure changes between the conditions. Given that the potential benefits of acute L-carnitine supplementation are still uncertain in the literature, additional research with larger sample sizes, including both male and female participants, and evaluating both acute and chronic L-carnitine supplementation with carbohydrate co-ingestion, is needed to clarify these findings in CrossFit^®^ athletes. These findings pertain to trained male CrossFit^®^ athletes and should not be assumed to extend to women without targeted investigation.

## Figures and Tables

**Table 1:** Differences in CrossFit^®^ performance between sessions and treatment conditions (supplement type).

Subject ID	Carnitine (Repetitions, *n*)	Placebo (Repetitions, *n*)	Δ (Carnitine-Placebo), Reps	%Δ vs. Placebo
A16	165	45	120	266.67
A11	303	240	63	26.25
A12	272	245	27	11.02
A18	181	155	26	16.77
A07	240	216	24	11.11
A06	47	32	15	46.88
A17	217	210	7	3.33
A03	230	230	0	0.00
A19	255	255	0	0.00
A14	255	255	0	0.00
A13	181	182	−1	−0.55
A05	137	138	−1	−0.72
A20	125	127	−2	−1.57
A15	300	303	−3	−0.99
A09	225	240	−15	−6.25
A08	226	250	−24	−9.60
A01	77	108	−31	−28.70
A10	285	330	−45	−13.64
A02	180	230	−50	−21.74
A04	197	250	−53	−21.20

## Data Availability

The data from this study can be obtained by contacting the corresponding author, subject to privacy and ethical considerations.
